# Global and Current Research Trends of Unilateral Biportal Endoscopy/Biportal Endoscopic Spinal Surgery in the Treatment of Lumbar Degenerative Diseases: A Bibliometric and Visualization Study

**DOI:** 10.1111/os.13216

**Published:** 2022-03-16

**Authors:** Pei‐lin Chu, Tao Wang, Jia‐le Zheng, Chong‐qing Xu, Yin‐jie Yan, Qing‐shan Ma, Yin Meng‐chen, Tian Da‐sheng

**Affiliations:** ^1^ Department of Orthopaedics Maanshan General Hospital of Ranger‐Duree Healthcare Anhui China; ^2^ Department of Orthopaedics The Second Hospital of Anhui Medical University Anhui China; ^3^ Department of Orthopaedics Longhua Hospital Shanghai University of Traditional Chinese Medicine Shanghai China

**Keywords:** Bibliometric analysis, Research trends, Spinal surgery, UBE/BESS, Visualization

## Abstract

The study aimed to make a bibliometric analysis of the current research situation in unilateral biportal endoscopy/biportal endoscopic spinal surgery (UBE/BESS). Research data sets were acquired from the Web of Science database. The study chosed “biportal endoscopic spinal surgery” OR “two portal endoscopic spinal surgery” OR “percutaneous biportal endoscopic decompression” OR “unilateral biportal endoscopy” OR “irrigation endoscopic discectomy” as the search terms. The literature search was limited to articles published before March 5, 2021. We only included original articles and reviews. VOS viewer and Citespace software were used to analyze the data and generate visualization knowledge maps. Annual trend of publications, distribution, H‐index status, co‐authorship status, and research hotspots were analyzed. A total of 74 publications met the requirement. The sum number of citations was 31,204, in which 19,336 were no self‐citations. The average citation of all the papers was 21.84 times. The H‐index of all the publications was 85. South Korea's total number of articles was far higher than that of other countries and regions (61, 82.4%), followed by United Arab Emirates, Egypt, and Peoples Republic of China (three, ranking second, accounting for 12.2% of the total). For the most productive authors, Choi ranked first with 21 articles, Kim ranked second with 16 articles, and Heo ranked third with 12 articles. The journal with the greatest number of publications was *World Neurosurgery*, with a total of 18 (39.1%) papers. *Clinics in Orthopedic Surgery* ranked second with six (13.0%) papers. In third place, there were fix articles published by *Asian Spine Journal* and *Neurospine*, accounting for 21.8% of the total articles. These top three journals accounted for 73.9% of all the papers. Spondylolisthesis and endoscopic decompression were the research hotspots in recent years. The number of publications has showed an upward trend with a stable rise in recent years. South Korea is the country with the highest productivity, not only in quality, but also in quantity. Barun Hosp and Leon Wiltse Mem Hosphave published most articles. Choi is the most productive author. *World Neurosurgery* is the most productive journal. Spondylolisthesis and endoscopic decompression are the research hotspots in recent years. Indeed, this study provides new insight into the growth and development of UBE/BESS.

## Graphical abstract

This study is to understand overall development trend of UBE. The number of publications showed an upward trend with a stable rise in recent years. Spondylolisthesis and endoscopic decompression are the research hotspots.

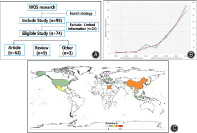

## Introduction

With the change of human lifestyle in modern society, the prevalence of lumbar degenerative diseases has increased^1^, ^2^, ^3^. Degenerative lumbar spinal diseases have become a common health problem and the most frequent indication for spinal surgery in individuals over 60 years. Traditionally, open discectomy and the decompression procedure have been the most common techniques for lumbar disc herniation and stenosis (without degenerative instability and spondylolisthesis). However, when a tubular approach is used in a microscopic setting, the ability to hand instruments might be restricted along with the vision. Percutaneous endoscopic surgery is one of the most common procedures for LDH and lumbar spinal stenosis. Whether the transforaminal or the interlaminar approach is used, endoscopic spinal surgery is performed through a single portal involving light source, irrigation, visualization, and instrumentation.

Despite use of a microscope or full‐endoscope, visualization is restricted and there are also technical difficulties that may be encountered by surgeons, which are particularly relevant in severe stenosis or in cases in need of bilateral decompression. Since the technique of unilateral biportal endoscopy/ biportal endoscopic spinal surgery (UBE/BESS) was first proposed and reported by De Antoni in 1996, adhering to the principle of precision and being minimally invasive, it could complete central spinal canal decompression, lateral recess decompression, and interbody bone graft fusion^4^, ^5^, ^6^. UBE/BESS was a new method that combined the advantages of interlaminar endoscopy and microscopic surgery. The use of the uniportal system was limited because of the combined channel (viewing and instrumental) that limited the independent movement of instruments. By contrast, the UBE/BESS system used independent channels for instruments; thus, movements were not restricted. Furthermore, instruments for both 30° or 0° arthroscopy for the knees and shoulders and standard laminectomy were used and additional devices were no longer needed. Moreover, the endoscopic trajectory was the same as that in conventional operation; thus, an experienced microscopic spine surgeon could achieve the necessary surgical skills without a steep learning curve. Under the consistent exploration and research of Korean scholars, the application of the technique has been continuously expanded to various spinal‐related diseases and satisfactory clinical results have been achieved. The new endoscopic technique approach has been applied to conventional arthroscopic systems for spinal disease.

UBE/BESS can be broadly divided into interlaminar and transforaminal approaches, both of which are performed under general anesthesia with the patient in the prone position on a radiolucent frame. The basic spine instruments include a Kerrison punch and a 0° or 30° 4‐mm arthroscope. Bipolar radiofrequency is used for hemostasis and an arthroscopic burr and a shaver are used to dissect and remove the bony and soft tissues. Compared with traditional surgery, UBE/BESS technology has its obvious advantage of being minimally invasive, but it also has inherent shortcomings, including long learning curve and lack of large sample data to support clinical safety and efficacy^7^, ^8^, ^9^, ^10^, ^11^, ^12^.

Bibliometrics is a type of analysis method regarding both quantity and quality, using mathematics, statistics, philology, and other professional knowledge and methods to comprehensively analyze the distribution of research results. One of the measures employed for this analysis includes citation frequency, which relates to the number of times the article is cited by researchers. Therefore, bibliometric analysis has been a mature tool to quantify the characteristics and scholarly impact of a specific field and can be applied vastly to assess the merits of a specific field and provide great insights to the growth and development of a subject^13^, ^14^.

In the past several years, this method has been widely used in various research areas. Citation analysis is the main methodology of bibliometric analysis. A citation is that one article uses another as a reference. The number of citations is not only an indicator of the impact of an article on the scientific community but also forms the basis of journal impact factor (IF) generation. Bibliometrics, although not an infallible technique, could serve as a valuable tool for directing the allocation of resources by funding agencies and for identifying potential of research areas in a discipline.

Although UBE/BESS has not yet become popular, it has attracted much interest. However, as far as we know, there has not been any bibliometric study about the trend of published articles of UBE/BESS. This research aimed to: (i) assess the characteristics of national productivity; (ii) visually present the research framework and overall knowledge structure; and (iii) provide the status and frontier trends of UBE/BESS.

## Materials and Methods

### Search Strategy and Refined Data

The data was collected from the Web of Science (WOS) database. WOS has a strict evaluation process, so it is a widely accepted tool for the subsequent bibliometric analysis. We chose “biportal endoscopic spinal surgery” OR “two portal endoscopic spinal surgery” OR “percutaneous biportal endoscopic decompression” OR “unilateral biportal endoscopy” OR “irrigation endoscopic discectomy” as the search terms. The literature search was limited to articles published before March 5, 2021. We only included original articles and reviews, while excluding basic research articles, editorial material, letters, and corrections. Two independent researchers were asked to review and evaluate the cited articles to guarantee the accuracy of the research. All different points were discussed until we reached agreements.

### Data Analysis

The collected data was imported into the Microsoft Excel 2017. It was analyzed for the annual trends of publications, distribution, citation, H‐Index status, co‐authorship status, research hotspots, and co‐citation status of the published paper in terms of quantity and quality. We used SPSS 20.0 to perform the statistical analyses and the statistical significance was considered at *p* < 0.05. We also used VOS viewer and Citespace software to create visualized pictures by the statistical results mentioned above.

## Results

### Current Status and Annual Trend of Study

**Figure 1:**
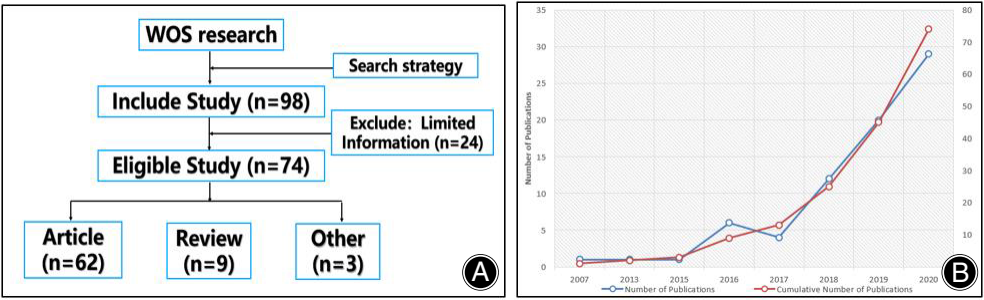
(A) Flow chart; (B) The annual trends of publications

We finally collected 74 articles from the WOS according to the inclusion criteria, including nine reviews and 65 original articles. Figure 1A showed the selection flow chart. The number of citations was 31,204, in which 19,336 were no self‐citations. The average citation of all the papers was 21.84 times. The H‐index of all the publications was 85.

Figure 1B showed the annual trends of publication numbers. In general, the total number of articles published about UBE/BESS technology was not large but tended to increase in a straight line since 2007 (one article), except that the number of papers published in 2017 decreased slightly (four articles). The number of published papers reached the highest level by 2020, and the total number of published papers reached 20. The results indicated that scientific researchers paid more attention to the field of UBE/BESS.

### The Distribution and Co‐Authorship Analysis of Countries

From the picture, we can see that a total of 13 countries or regions paid attention to the research of UBE/BESS. There are eight countries with a total volume of more than one article. South Korea's total number of articles was far higher than that of other countries and regions (61, 82.4%), followed by the United Arab Emirates, Egypt, and the Peoples Republic of China (three, ranking second, accounting for 12.2% of the total). Thailand, Singapore, Mexico, and Japan posted two articles, ranking third, accounting for 10.9% of the total. The number of citations could reflect the quality of a paper.

**Figure 2:**
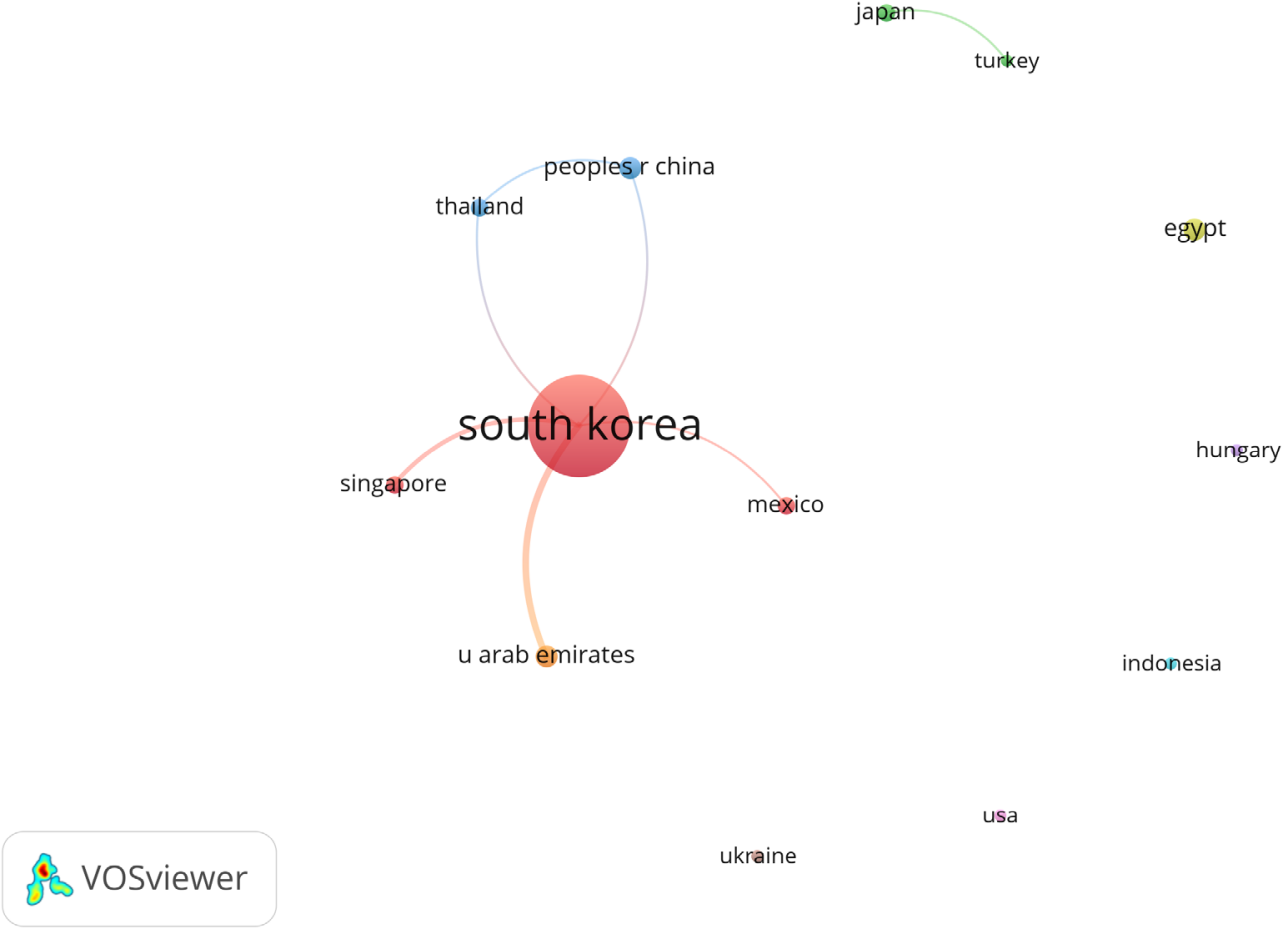
The country co‐authorship network of publications

H‐index is a reliable and authentic parameter for academic evaluation of core scientists. Because the largest number of documents is published in Korea, the number of citations was also the highest, which is also more than the sum of other regions (594). In addition, the number of citations of Egypt ranked second (48), followed by Thailand (29). South Korea rank first in H index (13), followed by United Arab Emirates (two), Egypt (two), and Thailand (two). Peoples Republic of China (one), Singapore (one), Mexico (one), USA (one), and Indonesia (one) ranked third. The results showed that South Korea was the country with the most published literature, not only in quantity, but also in quality. It could be concluded that South Korea had very in‐depth research in the field of UBE/BESS technology compared with other countries and regions. Citespace viewer software was employed to analyze the network visualization of co‐authorship relationship. Among the 13 countries’ studies about UBE/BESS, South Korea was the research center and maintained close cooperation with the Peoples Republic of China, Thailand, Singapore, Mexico, and United Arab Emirates. However, the cooperation between other countries was relatively weak (Figure 2).

### Distribution and Co‐Authorship Analysis of Institutions

**Figure 3:**
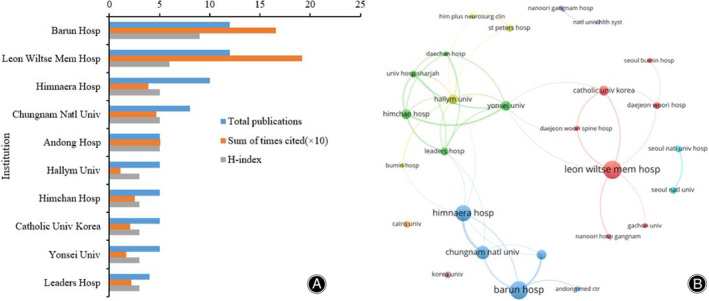
(A) The publication number, H‐index, and cited times of the top 10 institutes; (B) The institute co‐authorship network of publications

A total of 10 institutions contributed to the research on UBE/BESS (Figure 3A). The number of articles published by all institutions was more than three. Both Barun Hospital and Leon Wiltse Mem Hospital ranked the first (12), followed by Himnaera Hospital, which ranked second, and Chungnam Natational University which ranked third. The H‐index of cited time of Barun Hospital ranked first (nine), followed by Leon Wiltse Memorial Hosp (eight). Himnaera Hospital, Chungnam National University, and Andong Hospital ranked third (five). However, Leon Wiltse Memorial Hospital ranked the first in sum of times cited (19.2), followed by Barun Hospital (16.6) and Andong Hospital (5.1). Only institutes with a minimum of two articles were included in co‐authorship analysis. Twenty‐seven institutes met the threshold and were selected for analysis. It showed that Leon Wiltse Memorial Hospital, Chungnam National University and Hallym University had closely collaborated with their affiliated hospitals and research centers. But generally speaking, the cooperative relationship among different agencies were relatively close (Figure 3B).

### The Distribution and Co‐Authorship Analysis of Authors

**Figure 4:**
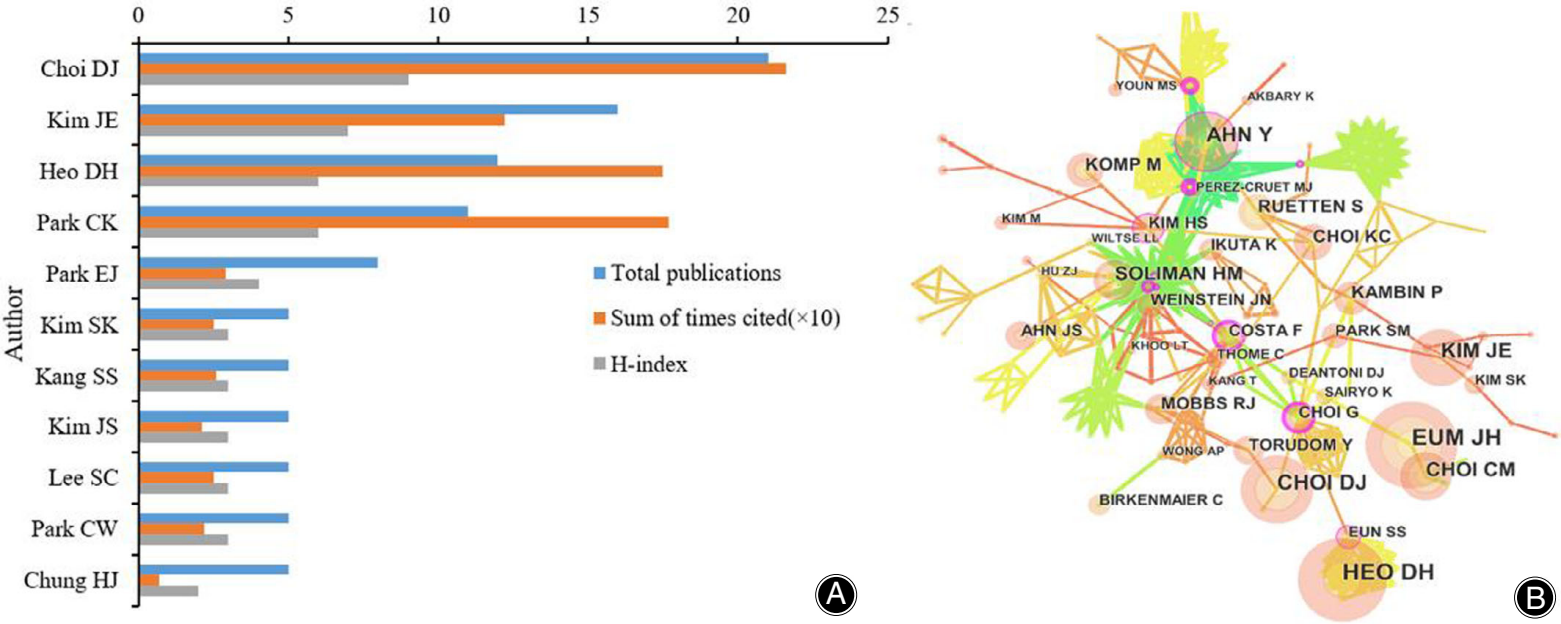
(A) The publication number, H‐index, and cited times of the top 10 authors; (B) The authors co‐authorship network of publications

A total of 11 authors published in the field of UBE/BESS technology research were retrieved, and the top five authors published more than five articles (Figure 4A). Choi DJ ranked the first with 21 articles, Kim JE ranked the second with 16 articles, and Heo DH ranked the third with 12 articles. The cited time of Choi DJ ranked the first (21.6), and his H index also ranked the first. The cited time of Park CK ranked the second (17.7), followed by Heo DH (17.5). The H‐index of Kim JE's H index ranked the second of all the authors (16), followed by Heo DH (six) and Park CK (six). Only authors who published a minimum of three articles were included. Thirty authors met the threshold and were selected for analysis. It showed that authors in the same country had relatively close collaboration. Nevertheless, the cooperation among authors from different countries was weak (Figure 4B).

### Distribution and Co‐Authorship Analysis of Published Journals

**Figure 5:**
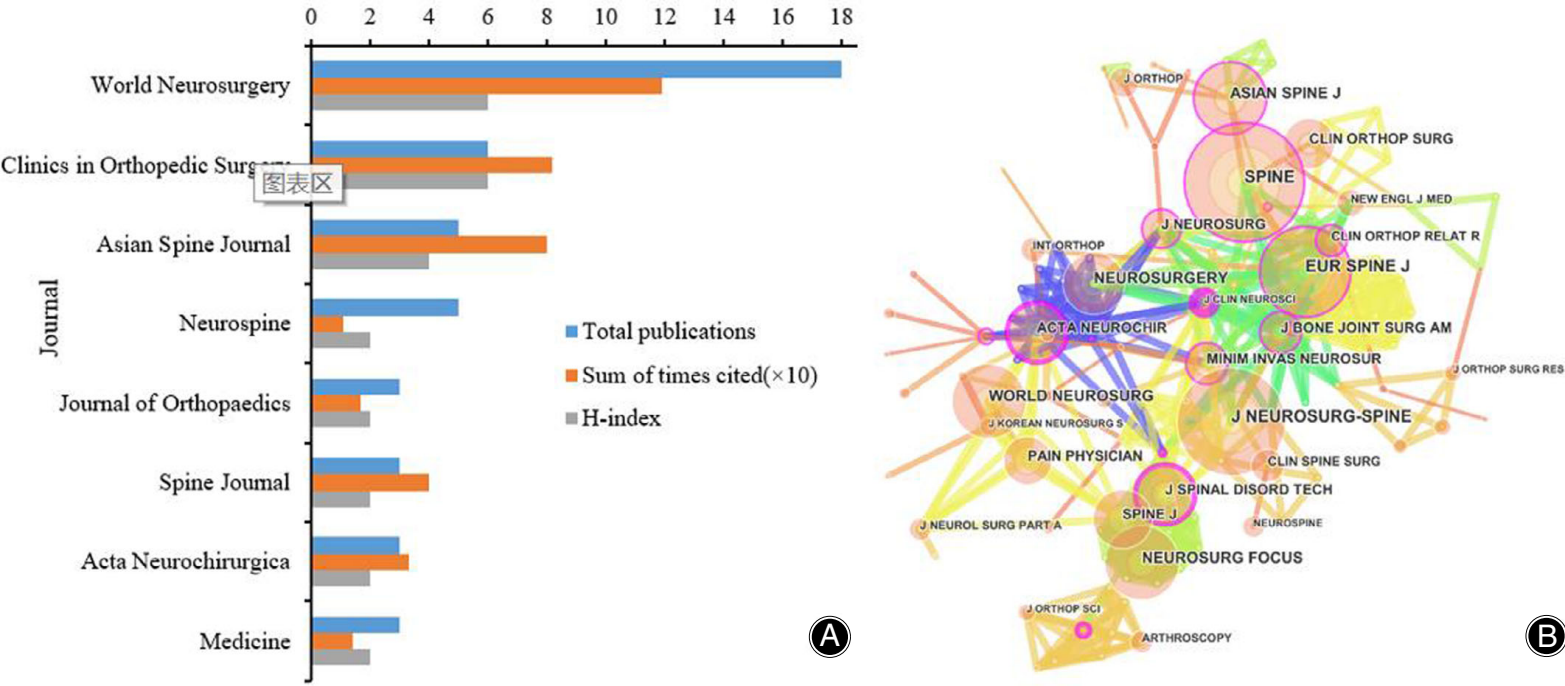
(A) The publication number, H‐index, and cited times of the top 10 journals; (B) The journals co‐authorship network of publications

All publications were published in eight journals which were shown in Figure 5A. The journal with the greatest number of publications was *World Neurosurgery* with a total of 18 (39.1%) papers. *Clinics in Orthopedic Surgery* ranked the second with six (13.0%) papers. In third place, there were five articles published by *Asian Spine Journal* and *Neurospine*, accounting for 21.8% of the total. These top three journals accounted for 73.9% of all the papers. Only 12 (26.1%) journals published more than three papers. In terms of the H‐index, both *World Neurosurgery* and *Clinics in Orthopedic Surgery* ranked first (six), followed by (19) *Asian Spine Journal*. *World Neurosurgery* (11.90) ranked first on the cited times as well, followed by *Clinics in Orthopedic Surgery* (8.20) and *Asian Spine Journal* (8.00). Figure 5B showed the co‐authorship relationship of journals. All of the included journals, *Spine*, *Neurosurgeryspine*, and *Eurospine* were in the center of research. In general, cooperation between journals was relatively strong. Table 1 shows the top 10 cited articles in terms of title, journal, authors, years, and citation numbers^10^, ^20^, ^26^, ^32^, ^33^, ^34^, ^35^, ^36^, ^37^, ^38^. The first literature was cited 35 times, and the least cited 12 times. Among the top 10 citations, the published magazines were scattered, and only two articles were published in *Neurosurgeryspine*.

**Table 1:** Top 10 cited articles in the field of UBE/BESS

Rank	Title	Journal	Authors	Years	Freq
1	Biportal Endoscopic Lumbar Decompression for Lumbar Disk Herniation and Spinal Canal Stenosis: A Technical Note.	J NEUROSURG‐SPINE	EUM JH	2016	35
2	Fully endoscopic lumbar interbody fusion using a percutaneous unilateral biportal endoscopic technique: technical note and preliminary clinical results.	NEUROSURG FOCUS	HEO DH	2017	22
3	Learning Curve Associated with Complications in Biportal Endoscopic Spinal Surgery: Challenges and Strategies.	ACTA NEUROCHIR	CHOI CM	2016	21
4	Biportal Endoscopic Spinal Surgery for Recurrent Lumbar Disc Herniations.	CLIN ORTHOP SURG	CHOI DJ	2016	19
5	Can Percutaneous Biportal Endoscopic Surgery Achieve Enough Canal Decompression for Degenerative Lumbar Stenosis? Prospective Case–Control Study.	WORLD NEUROSURG	HEO DH	2018	14
6	Irrigation endoscopic decompressive laminotomy. A new endoscopic approach for spinal stenosis decompression.	SPINE J	SOLIMAN HM	2015	13
7	Indirect foraminal decompression after anterior lumbar interbody fusion: a prospective radiographic study using a new pedicle‐to‐pedicle technique.	J NEUROSURG‐SPINE	MOBBS RJ	2014	13
8	Growth of Asymptomatic Intracranial Fusiform Aneurysms: Incidence and Risk Factors.	CLIN ORTHOP SURG	KIM JE	2018	12
9	Two Portal Percutaneous Endoscopic Decompression for Lumbar Spinal Stenosis: Preliminary Study.	ASIAN SPINE J	TORUDOM Y	2016	12
10	Bilateral spinal decompression of lumbar central stenosis with the full‐endoscopic interlaminar *versus* microsurgical laminotomy technique: a prospective, randomized, controlled study.	PAIN PHYSICIAN	KOMP M	2015	12

### The Keyword Analysis of Research Hotspots

**Figure 6:**
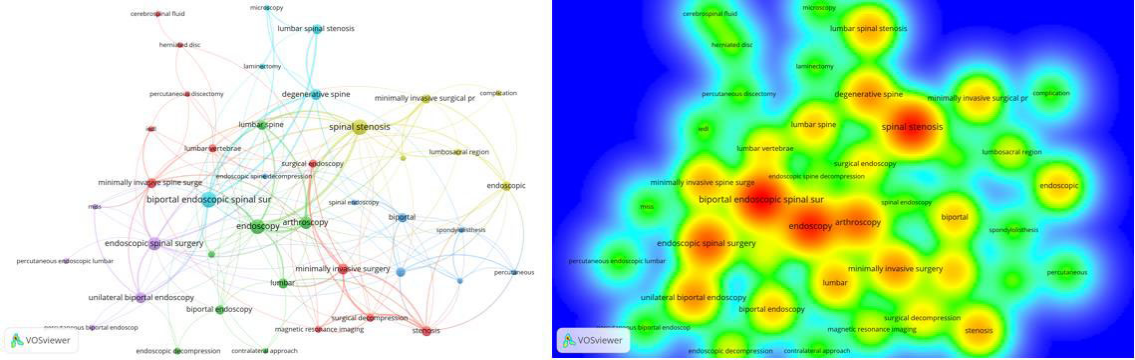
(A) Keywords co‐occurrence network of publications; (B) Keywords density visualization map of publications

We imported the data of keywords into VOS viewer to create visualized pictures of keyword co‐occurrence, which could reflect the research hotspots effectively. Figure 6 showed the keywords and research focuses related to UBE/BESS. The bigger nodes and darker color showed a larger weight of the keyword. Forty‐two keywords formed a relatively scattered cluster. But after combing, they could be divided into three categories: one was the indication for UBE/BESS technology, including spinal canal stenosis and degeneration; the second was for the technology itself, focusing on its minimally invasive quality; and the third was to pay attention to the development of UBE/BESS technology.

**Figure 7:**
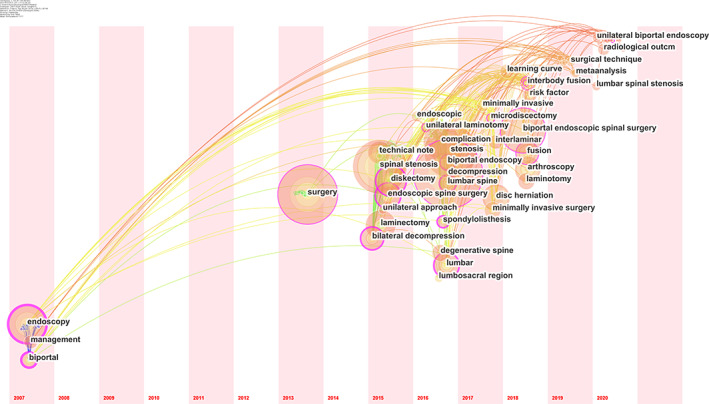
The keywords timeline view of publications

Table 2 showed 10 meaningful keywords with the largest number of citations. The red and blue bars respectively presented the frequently and infrequently cited keywords. Figure 7 showed the keywords timeline view of publications, which presented the research frontiers. From a minimally invasive point of view, the research on the indications of UBE/BESS technology increasingly became the focus of research, including spondylolisthesis and endoscopic decompression.

**Table 2:** Top 15 keywords with the strongest citation bursts

Keywords	Strength	Begin	End	2007–2020
Transcallosal microsurgery	0.6609	2007	2012	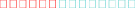
Biportal	0.7817	2007	2017	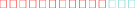
Neuroendoscopy	0.6609	2007	2012	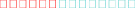
Rigid endoscope	0.6609	2007	2012	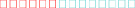
Surgical consideration	0.6609	2007	2012	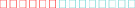
Transventricular approach	0.6609	2007	2012	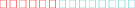
Neuronavigation	0.6609	2007	2012	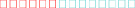
Percutaneous discectomy	0.6824	2008	2014	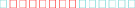
Endoscopic discectomy	0.6824	2008	2014	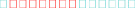
Lumbar discectomy	0.6824	2008	2014	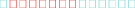
Minimally invasive spine surgery	0.6824	2008	2014	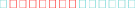
Spondylolisthesis	0.6993	2016	2017	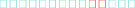
Endoscopic decompression	0.6865	2016	2018	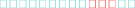
Lumbosacral region	1.0329	2016	2018	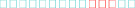
Minimally invasive surgery	1.0341	2017	2020	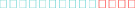

## Discussion

### Characteristics of National Productivity of UBE/BESS

There has been consistent development of minimally invasive technology in the past decades. In order to clarify the important role of UBE/BESS technology in the treatment of spinal surgical diseases and to promote the development of medicine, we made a quantitative analysis and description of the published literature by using the method of bibliometrics in the paper and analyzed each article on the basis of a comprehensive search of UBE/BESS. By the method of bibliometrics, this paper made statistics, induction, collation and analysis of the time distribution, regional distribution, periodical distribution, institution, author, and research type. In our study, we drew the knowledge graph, systematically combed the current research situation in the field of UBE/BESS technology, and analyzed the academic development trend.

Since the start of UBE/BESS technology, the overall number of published studies had shown a steady growth trend, which indicated that UBE/BESS technology research had entered a stage of rapid development and maintained a high degree of heat. As a new surgical technique, UBE/BESS had a short history and less published literature^7^, ^15^, ^16^, ^17^, ^18^. Literature research showed that only 13 countries and regions had paid attention to it. In addition, 82.4% of the literature published came from South Korea, and other leading countries included China, Egypt, and so on.

The number of citations could reflect the quality of the literature, and H index was the most reliable and true parameter for academic evaluation of core scientists. Data research showed that South Korea had the most academic contribution in the field of UBE/BESS, as both the total number of published literature and H index were the highest. South Korea occupied a dominant position in the field of UBE/BESS technology research and led the direction of scientific research. For the other countries and regions, they should continue to increase financial investment, accelerate the improvement of scientific research, and shorten the gap in medical research with other countries and regions.

### Research Framework and Overall Knowledge Structure of UBE/BESS

Regarding the research on UBE/BESS technology, the number of studies published by Barun Hospital and Leon Wiltse Memorial Hospital was twice as much as that of other research institutions. Both scholars were from South Korea, their H index ranked first and second, respectively, and their sum of times cited was also in the top two positions, which was enough to prove the advanced UBE/BESS technology in South Korea. The center of UBE/BESS technology research in the world was in South Korea. Only by constantly strengthening medical exchanges with South Korea could we go further and further on the development of UBE/BESS technology research. Choi DJ, who had high academic influence, ranked first in the number of articles and focused on the exploration of UBE/BESS technology in South Korea^19^, ^20^, ^21^, ^22^, ^23^, ^24^.

We paid full attention to the advantages of bibliometrics, the degree of communication and cooperation among researchers through co‐authored research, to know clearly the direction of research and development. The data showed that the technical cooperation of UBE/BESS was relatively scattered, the technology popularization rate was low, and there was a large gap in the technological level of different countries. Korean scholars continued to develop UBE/BESS technology, covering lumbar, cervical, and thoracic diseases. Therefore, it was urgent to strengthen academic exchanges and cooperation among countries, regions, and institutions, and further formed an academic cooperation atmosphere in order to promote the steady development of scientific research^16^, ^21^, ^22^, ^25^, ^26^, ^27^.

### Research Status and Frontier Trends of UBE/BESS

It was a good choice for most spinal surgeons to solve the pain and alleviate the disease by traditional surgery. However, as the minimally invasive concept of spinal surgery gained popularity, how to reduce the negative impact of traditional open surgery and achieve the accuracy of surgery also become the focus of clinical research. Due to the iatrogenic destruction of the posterior structure of the spine, traditional surgery may lead to postoperative pain, muscle atrophy, iatrogenic spinal instability, adjacent segmental degeneration, and perioperative blood loss. Therefore UBE/BESS technology was based on the concept of being minimally invasive. Endoscopic treatments focused on reducing the negative effects on muscles, ligaments, and other anatomical structures. It could reduce intraoperative trauma, the amount of bleeding, and shorten the recovery period after operation^12^, ^18^, ^26^, ^27^, ^28^, ^29^, ^30^, ^31^.

With the expansion of the concept of of being minimally invasive, the consistent research, development of minimally invasive devices, and the accumulation of clinical experience, the clinical application of UBE/BESS technology would become more and more extensive, the curative effect would be better, and learning curve would be much flatter; therefore, it would have a bright future^32^, ^33^, ^34^. It not only relieved the pain of patients, but also could provide new treatment ideas and directions for medical practitioners. However, UBE/BESS required high surgical techniques, rich spatial imagination, and proficiency in percutaneous puncture^20^, ^35^, ^36^, ^37^, ^38^. At present, high‐quality studies were mainly focused on the treatment of lumbar spinal stenosis compared with microscope, and there was a lack of a high‐quality clinical comparative study of single‐channel endoscopy surgeries. In a word, no matter which kind of surgical methods are used to treat surgical diseases, clinicians should closely combine their own surgical skills with the characteristics of the disease and obtain the best clinical effect through minimum injury.

### Strengths and Limitations

In this study, we made a qualitative and quantitative analysis of the literature in the field of UBE/BESS by bibliometrics analysis and visualization tools. In order to ensure the objectivity and comprehensiveness of the research, we conducted a systematic literature search on WOS, collected convincing data of many aspects to obtain a high degree of recognition. However, there are still some limitations to this study. First, the data analysis of bibliometrics only includes the published literature in WOS database, not the unpublished and non‐English literature. Secondly, bibliometrics data change with the passage of time because the selected publication period is from 1990 to 2020, and some recently published high‐quality literature may not be cited frequently because of the short time of publication, which may lead to some differences between the research results and the real situation. Thirdly, all citations are included in the study without quality screening, whether because of its positive contribution or because of its negative impact or poor quality.

## Conclusions

This study provided a global overview of literature, researchers, research institutions, and research interests on UBE/BESS. The number of published studies showed an upward trend overall; South Korea was the leader in this field and has the greatest influence. Buran Hospital and Leon Wiltse Mem Hospital made the greatest contribution in this field. Spondylolisthesis and endoscopic decompression were the research hotspots in recent years. Through this study, it has been made clear that the research activities of UBE/BESS in the world have provided a new direction for the development of scientific research.

## Data Availability

The datasets generated during and/or analyzed during the current study are publicly available.

## References

[1] Hu QF , Pan H , Fang YY , Jia GY . Percutaneous endoscopic lumbar discectomy for high‐grade down‐migrated disc using a trans‐facet process and pedicle‐complex approach: a technical case series. Eur Spine J. 2018, 27: 393–402. 29119334 10.1007/s00586-017-5365-3

[2] Yang JC , Kim SG , Kim TW , Park KH . Analysis of factors contributing to postoperative spinal instability after lumbar decompression for spinal stenosis. Korean J Spine. 2013, 10: 149–54. 24757477 10.14245/kjs.2013.10.3.149 PMC3941765

[3] Hu ZJ , Fang XQ , Zhou ZJ , Wang JY , Zhao FD , Fan SW . Effect and possible mechanism of muscle‐splitting approach on multifidus muscle injury and atrophy after posterior lumbar spine surgery. J Bone Joint Surg Am. 2013, 95: 191–9. 10.2106/JBJS.L.01607 24352778

[4] Aygun H , Abdulshafi K . Unilateral Biportal endoscopy versus tubular microendoscopy in Management of Single Level Degenerative Lumbar Canal Stenosis: a prospective study. Clin Spine Surg. 2021, 34: 323–8. 10.1097/BSD.0000000000001122 PMC8225231 33470660

[5] Kim JE , Choi DJ . Unilateral biportal endoscopic decompression by 30° endoscopy in lumbar spinal stenosis: technical note and preliminary report. J Orthop. 2018, 15: 366–71. 29881155 10.1016/j.jor.2018.01.039 PMC5990374

[6] Park JH , Jun SG , Jung JT , Lee SJ . Posterior percutaneous endoscopic cervical Foraminotomy and Diskectomy with unilateral Biportal endoscopy. Orthopedics. 2017, 40: 779–83. 10.3928/01477447-20170531-02 28585996

[7] Pranata R , Lim MA , Vania R , July J . Biportal endoscopic spinal surgery versus microscopic decompression for lumbar spinal stenosis: a systematic review and meta‐analysis. World Neurosurg. 2020, 138: e450–8. 32147545 10.1016/j.wneu.2020.02.151

[8] Heo DH , Lee DC , Park CK . Comparative analysis of three types of minimally invasive decompressive surgery for lumbar central stenosis: biportal endoscopy, uniportal endoscopy, and microsurgery. Neurosurg Focus. 2019, 46: E9. 10.3171/2019.2.FOCUS197 31042664

[9] Yue JJ , Long W . Full endoscopic spinal surgery techniques: advancements, indications, and outcomes. Int J Spine Surg. 2015, 9: 17. 26114086 10.14444/2017 PMC4480053

[10] Soliman HM . Irrigation endoscopic decompressive laminotomy. A new endoscopic approach for spinal stenosis decompression. Spine J. 2015, 15: 2282–9. 26165475 10.1016/j.spinee.2015.07.009

[11] Hwa Eum J , Hwa Heo D , Son SK , Park CK . Percutaneous biportal endoscopic decompression for lumbar spinal stenosis: a technical note and preliminary clinical results. J Neurosurg Spine. 2016, 24: 602–7. 26722954 10.3171/2015.7.SPINE15304

[12] Kang T , Park SY , Kang CH , Lee SH , Park JH , Suh SW . Is biportal technique/endoscopic spinal surgery satisfactory for lumbar spinal stenosis patients?: a prospective randomized comparative study. Medicine (Baltimore). 2019, 98: e15451. 31045817 10.1097/MD.0000000000015451 PMC6504265

[13] Yin MC , Wang HS , Yang X , Xu CQ , Wang T , Yan YJ , et al. A bibliometric analysis and visualization of current research trends in Chinese medicine for osteosarcoma. Chin J Integr Med. 2020, 1–8. 10.1007/s11655-020-3429-4 32876857

[14] Yin M , Xu C , Mo W . The 100 Most cited articles on lumbar spinal stenosis: a bibliometric analysis. Global. Spine J. 2020, 2192568220952074: 219256822095207. 10.1177/2192568220952074 PMC9121157 32856488

[15] Ahn DK , Lee JS , Shin WS , Kim S , Jung J . Postoperative spinal epidural hematoma in a biportal endoscopic spine surgery. Medicine (Baltimore). 2021, 100: e24685. 33578600 10.1097/MD.0000000000024685 PMC10545396

[16] Lee HG , Kang MS , Kim SY , Cho KC , Na YC , Cho JM , et al. Dural injury in unilateral Biportal endoscopic spinal surgery. Global Spine J. 2021, 11: 845–51. 32762357 10.1177/2192568220941446 PMC8258823

[17] Kim JE , Yoo HS , Choi DJ , Park EJ , Hwang JH , Suh JD , et al. Effectiveness of gelatin‐thrombin matrix sealants (Floseal®) on postoperative spinal epidural hematoma during single‐level lumbar decompression using Biportal endoscopic spine surgery: clinical and magnetic resonance image study. Biomed Res Int. 2020, 2020: 4801641–10. 10.1155/2020/4801641 PMC7368184 32695815

[18] Kang T , Park SY , Lee SH , Park JH , Suh SW . Spinal epidural abscess successfully treated with biportal endoscopic spinal surgery. Medicine (Baltimore). 2019, 98: e18231. 31852084 10.1097/MD.0000000000018231 PMC6922448

[19] Choi CM , Chung JT , Lee SJ , Choi DJ . How I do it? Biportal endoscopic spinal surgery (BESS) for treatment of lumbar spinal stenosis. Acta Neurochir. 2016, 158: 459–63. 26782827 10.1007/s00701-015-2670-7 PMC4752582

[20] Choi DJ , Choi CM , Jung JT , Lee SJ , Kim YS . Learning curve associated with complications in Biportal endoscopic spinal surgery: challenges and strategies. Asian Spine J. 2016, 10: 624–9. 27559440 10.4184/asj.2016.10.4.624 PMC4995243

[21] Kim N , Jung SB . Percutaneous unilateral Biportal endoscopic spine surgery using a 30‐degree arthroscope in patients with severe lumbar spinal stenosis: a technical note. Clin Spine Surg. 2019, 32: 324–9. 31464695 10.1097/BSD.0000000000000876 PMC6791497

[22] Lin GX , Huang P , Kotheeranurak V , Park CW , Heo DH , Park CK , et al. A systematic review of unilateral Biportal endoscopic spinal surgery: preliminary clinical results and complications. World Neurosurg. 2019, 125: 425–32. 30797907 10.1016/j.wneu.2019.02.038

[23] Park SM , Kim HJ , Kim GU , Choi MH , Chang BS , Lee CK , et al. Learning curve for lumbar decompressive laminectomy in Biportal endoscopic spinal surgery using the cumulative summation test for learning curve. World Neurosurg. 2019, 122: e1007‐1007e1013. 30404053 10.1016/j.wneu.2018.10.197

[24] Song KS , Lee CW , Moon JG . Biportal endoscopic spinal surgery for bilateral lumbar Foraminal decompression by switching Surgeon's position and primary 2 portals: a report of 2 cases with technical note. Neurospine. 2019, 16: 138–47. 30943716 10.14245/ns.1836330.165 PMC6449833

[25] Heo DH , Kim JS , Park CW , Quillo‐Olvera J , Park CK . Contralateral sublaminar endoscopic approach for removal of lumbar Juxtafacet cysts using percutaneous Biportal endoscopic surgery: technical report and preliminary results. World Neurosurg. 2019, 122: 474–9. 30458327 10.1016/j.wneu.2018.11.072

[26] Choi DJ , Jung JT , Lee SJ , Kim YS , Jang HJ , Yoo B . Biportal endoscopic spinal surgery for recurrent lumbar disc Herniations. Clin Orthop Surg. 2016, 8: 325–9. 27583117 10.4055/cios.2016.8.3.325 PMC4987318

[27] Kim JE , Choi DJ . Unilateral Biportal endoscopic spinal surgery using a 30° arthroscope for L5‐S1 Foraminal decompression. Clin Orthop Surg. 2018, 10: 508–12. 30505421 10.4055/cios.2018.10.4.508 PMC6250961

[28] Kim JE , Choi DJ , Kim MC , Park EJ . Risk factors of postoperative spinal epidural hematoma after Biportal endoscopic spinal surgery. World Neurosurg. 2019, 129: e324–9. 31158548 10.1016/j.wneu.2019.05.141

[29] Choi DJ , Kim JE . Efficacy of Biportal endoscopic spine surgery for lumbar spinal stenosis. Clin Orthop Surg. 2019, 11: 82–8. 30838111 10.4055/cios.2019.11.1.82 PMC6389528

[30] Ahn JS , Lee HJ , Park EJ , Kim SB , Choi DJ , Kwon YS , et al. Multifidus muscle changes after Biportal endoscopic spinal surgery: magnetic resonance imaging evaluation. World Neurosurg. 2019, 130: e525–34. 31254694 10.1016/j.wneu.2019.06.148

[31] Ahn JS , Lee HJ , Choi DJ , Lee KY , Hwang SJ . Extraforaminal approach of biportal endoscopic spinal surgery: a new endoscopic technique for transforaminal decompression and discectomy. J Neurosurg Spine. 2018, 28: 492–8. 29473790 10.3171/2017.8.SPINE17771

[32] Eun SS , Eum JH , Lee SH , Sabal LA . Biportal endoscopic lumbar decompression for lumbar disk herniation and Spinal Canal stenosis: a technical note. J Neurol Surg A Cent Eur Neurosurg. 2017, 78: 390–6. 27652804 10.1055/s-0036-1592157

[33] Heo DH , Son SK , Eum JH , Park CK . Fully endoscopic lumbar interbody fusion using a percutaneous unilateral biportal endoscopic technique: technical note and preliminary clinical results. Neurosurg Focus. 2017, 43: E8. 10.3171/2017.5.FOCUS17146 28760038

[34] Rao PJ , Maharaj MM , Phan K , Lakshan Abeygunasekara M , Mobbs RJ . Indirect foraminal decompression after anterior lumbar interbody fusion: a prospective radiographic study using a new pedicle‐to‐pedicle technique. Spine J. 2015, 15: 817–24. 25543011 10.1016/j.spinee.2014.12.019

[35] Moon J , Cho YD , Yoo DH , Lee J , Kang HS , Cho WS , et al. Growth of asymptomatic intracranial fusiform aneurysms: incidence and risk factors. Clin Neuroradiol. 2019, 29: 717–23. 29777291 10.1007/s00062-018-0695-z

[36] Torudom Y , Dilokhuttakarn T . Two portal percutaneous endoscopic decompression for lumbar spinal stenosis: preliminary study. Asian Spine J. 2016, 10: 335–42. 27114776 10.4184/asj.2016.10.2.335 PMC4843072

[37] Komp M , Hahn P , Oezdemir S , Giannakopoulos A , Heikenfeld R , Kasch R , et al. Bilateral spinal decompression of lumbar central stenosis with the full‐endoscopic interlaminar versus microsurgical laminotomy technique: a prospective, randomized, controlled study. Pain Phys. 2015, 18: 61–70. 25675060

[38] Heo DH , Quillo‐Olvera J , Park CK . Can Percutaneous Biportal Endoscopic Surgery Achieve Enough Canal Decompression for Degenerative Lumbar Stenosis? Prospective Case‐Control Study. World Neurosurg X. 2018, 120: e684–9. 10.1016/j.wneu.2018.08.144 30165228

